# Differentiating allergic and irritant contact dermatitis by high-definition optical coherence tomography: a pilot study

**DOI:** 10.1007/s00403-014-1492-4

**Published:** 2014-09-04

**Authors:** Marc A. L. M. Boone, Gregor B. E. Jemec, V. Del Marmol

**Affiliations:** 1 Department of Dermatology, Hôpital Erasme, Université Libre de Bruxelles, 808 Route de Lennik, 1070 Brussels, Belgium; 2 Department of Dermatology, Roskilde Hospital, Health Sciences Faculty, University of Copenhagen, Copenhagen, Denmark

**Keywords:** High-definition optical coherence tomography, Patch test scoring, Transition metals, Allergic contact dermatitis, Irritant contact dermatitis

## Abstract

Differentiation of allergic contact dermatitis (ACD) and irritant contact dermatitis (ICD) is important because of different management requirements. Various non-invasive tests have been used in an attempt to improve diagnosis. In irritant dermatitis, thickening of the epidermis has been a constant finding. High-Definition Optical Coherence Tomography (HD-OCT) is a non-invasive real-time three-dimensional imaging technique with cellular resolution for which an adapted algorithmic method for pattern analysis discriminating inflammatory skin diseases has been proposed. The aim of this study was threefold. (1) To evaluate the correlation between HD-OCT features and clinical scores of allergic and irritant patch test reactions. (2) To explore the potential of HD-OCT in optimizing the visual patch test scoring. (3) To assess in vivo the cytological and 3-D micro-architectural differences in skin reaction types between doubtful positive ACD and ICD. Twenty-two volunteers were patch tested using potassium(VI)dichromate, cobalt(II)chloride, nickel(II) sulfate and palladium(II)chloride. Visual patch test scoring and HD-OCT assisted patch test scoring were performed at 48 and 96 h after patch test application according to ECDRG guidelines. Selected HD-OCT features correlated well with clinical severity scores. HD-OCT assessment improved the visual patch test scoring although not significantly. Increased epidermal thickness observed in ICD at first reading was a significant finding useful in differentiating doubtful (+?) ACD from irritant (IR) ICD reactions. In conclusion, HD-OCT might be a unique tool for in vivo non-invasive real-time three-dimensional epidermal thickness measurements helping to differentiate IR from doubtful (+?) reactions in patch testing. Selected HD-OCT features corresponded well with severity of visual scoring. These features might help to quantify the degree of inflammation in inflammatory skin conditions. HD-OCT might help in optimizing visual patch test scoring in some situations.

## Introduction

Contact dermatitis (CD) is an inflammatory eczematous skin disease mostly caused by chemicals or metal ions that exert toxic effects without inducing a T cell response (contact irritants) or by small reactive chemicals that modify proteins and induce innate and adaptive immune responses (contact allergens) [24]. Irritant and allergic contact dermatitis represent a major health problem. In general, irritant contact dermatitis (ICD) is more frequent than allergic contact dermatitis (ACD) [10]. The prevalence of CD is high as it is suggested that almost 20 % of the general population suffers from CD to at least one chemical, most commonly nickel (Ni) [19]. Differentiation of ACD and ICD is important because of different therapeutic and management options. In most cases contact dermatitis is suspected from anamnestic and clinical findings. Diagnosis is confirmed by patch testing. Recording of patch test reactions follows the guidelines of the International Contact Dermatitis Research Group as shown in Table 1 [33]. There is rarely disagreement concerning the reading of obvious irritant and ++ or +++ allergic reactions. By contrast the reading of +? and + reactions and some irritant reactions may cause difficulties. Skin biopsy is of little help in distinguishing the two because histopathologic changes and inflammatory infiltrate are often similar at this stage [21].

**Table 1:** Recording of patch test reactions according to the international Contact Dermatitis Research Group (ICDRG) [4]

+?	Doubtful reaction; faint erythema only
+	Weak positive reaction; erythema, infiltration, possibly papules
++	Strong positive reaction; erythema, infiltration, papules, vesicles
+++	Extreme positive reaction; intense erythema and infiltration and coalescing vesicles
−	Negative reaction
IR	Irritant reaction of different types
NT	Not tested

Different non-invasive tests have therefore been used in an attempt to improve diagnostic specificity [8, 11, 29, 30]. Among these, reflectance confocal microscopy (RCM) has been shown to be a promising tool for the differentiation of acute allergic and irritant contact dermatitis in vivo [31]. Relevant RCM features for diagnosis of ACD and ICD have been published recently [3]. Conventional optical coherence tomography (OCT) seems to be useful as an objective parameter in grading severity of patch test reactions [11]. Moreover, in irritant contact dermatitis thickening of the epidermis was a constant finding using different techniques and which could be monitored over time [34].

High-definition optical coherence tomography (HD-OCT) is a more recently introduced non-invasive three-dimensional imaging technique with cellular resolution [4–7]. An adapted algorithmic method for pattern analysis in inflammatory skin diseases has been suggested based on inflammation related HD-OCT features [7].

The aim of this pilot study is (1) to evaluate the correlation between selected HD-OCT features with clinical severity of patch test reactions (2) to explore the possible optimization of the visual patch test scoring of reactions to transition metals by HD-OCT and (3) to assess in vivo and real time the cytological and 3-D micro-architectural differences in skin reaction types of doubtful positive ACD and ICD.

## Materials and methods

### Patients and protocol design

Twenty-two Caucasian subjects with Fitzpatrick skin phototype II or III, aged between 18 and 69 years, were selected for this study. Inclusion criteria were (1) suspected contact allergy to transition metals (2) patch testing on the back using the European Standard Series including potassium dichromate (VI) Cr_2_K_2_O_7_ (in 0.5 % petrolatum), cobalt (II) chloride hexahydrate CoCl_2_^.^6H_2_O (1 % petrolatum) and Nickel(II) sulfate hexahydrate NiO_4_S^.^6H_2_O (5 % petrolatum) AND extended at least with palladium (II)chloride PdCl_2_ (2 % petrolatum) (Chemotechnique Diagnostics, Vellinge, Sweden). (3) Positive irritant or allergic reaction(s) at first and/or second reading to transition metal(s), (4) availability of good quality HD-OCT imaging of reactions at first and second reading, and (5) all subjects provided informed consent for HD-OCT imaging.

The selected transition metals are capable of inducing both ICD and ACD reactions and therefore were considered particularly suitable for this study. Tests were applied using Finn Chambers^®^ according to guidelines (SmartPractice, Phoenix, USA). The patch test substances were removed after 48 h and evaluated by clinical assessment and HD-OCT imaging 20 min after removal (first reading). A second reading was performed 48 h after removal of the patch (Table 2) Both readings are essential because of difference in kinetics of allergic and irritant contact reactions in vivo [4, 19]. A control site of normal skin and a site exposed to petrolatum (vehicle) only were also evaluated. Clinical photographs of skin reactions were taken under standardized conditions with a digital camera.

**Table 2:** Visual (V) and HD-OCT assisted (H) patch test scoring

# Subject	First reading after 48 H								Second reading After 96 H							
	Cr		Co		Ni		Pd		Cr		Co		Ni		Pd	
	V	H	V	H	V	H	V	H	V	H	V	H	V	H	V	H
1	–	–	–	–	+?	+?	+?	+?	–	–	–	–	+/++	+/++	+	+
2	+?	+?	IR	IR	–	–	–	–	–	–	IR	IR	–	–	–	–
3	IR	IR	IR	IR	–	–	–	–	IR	IR	IR	IR	–	–	**IR**	**+?**
4	++	++	++	++	+	+	–	–	++	++	++	++	+	+	–	–
5	–	–	–	–	IR	IR	–	–	–	–	–	–	+	+	**IR**	**+?**
6	+*?*	*IR*	–	–	–	–	–	–	IR	IR	–	–	IR	IR	IR	IR
7	IR	IR	IR	IR	–	–	–	–	–	–	IR	IR	–	–	–	–
8	–	–	–	–	+	+	–	–	–	–	–	–	+	+	–	–
9	–	–	+	+	+++	+++	+	+	–	–	+	+	+++	+++	+	+
10	–	–	IR	IR	–	–	–	–	–	–	IR	IR	–	–	–	–
11	IR	IR	IR	IR	+?	+?	+*?*	*IR*	IR	IR	IR	IR	+	+	IR	IR
12	IR	IR	IR	IR	IR	IR	–	–	IR	IR	IR	IR	IR	IR	–	–
13	IR	IR	IR	IR	IR	IR	–	–	IR	IR	IR	IR	–	–	IR	IR
14	–	–	IR	IR	–	–	–	–	–	–	–	–	+*?*	*IR*	–	–
15	–	–	–	–	+?	+?	–	–	–	–	–	–	+	+	+?	+?
16	IR	IR	–	–	+	+	IR	IR	–	–	–	–	+	+	–	–
17	IR	IR	–	–	–	–	–	–	IR	IR	–	–	**IR**	**+?**	–	–
18	IR	IR	–	–	–	–	–	–	IR	IR	–	–	–	–	**IR**	**+?**
19	IR	IR	–	–	+/++	+/++	+?	+?	–	–	–	–	++	++	**IR**	**+**
20	–	–	IR	IR	+	+	+	+	–	–	+*?*	*IR*	++	++	+	+
21	–	–	–	–	+	+	IR	IR	–	–	–	–	+/++	+/++	IR	IR
22	IR	IR	IR	IR	IR	IR	–	–	IR	IR	IR	IR	IR	IR	–	–
Irr/All ratio	10/3	11/2	10/2	10/2	4/10	4/10	2/5	3/4	8/1	8/1	8/3	9/2	4/12	4/12	8/4	4/8
	First reading								Second reading							
	Total number of reactions				46				Total number of reactions				48			
	Irritative reactions visual				26				Irritative reactions visual				28			
	Irritative reactions HD-OCT				28				Irritative reaction HD-OCT				25			
	Allergic reactions visual				20				Allergic reactions visual				20			
	Allergic reactions HD-OCT				18				Allergic reactions HD-OCT				23			
	Misclassification +? → IR^a^				2				Misclassification +? → IR^a^				2			
	Misclassification IR → +?^b^				0				Misclassification IR → +?^b^				5			

^a^Italic misclassified lesions: visual (+?) → HD-OCT (IR)

^b^Bold misclassified lesions: visual (IR) → HD-OCT (+?)

All clinical and HD-OCT assessments have been performed by the same investigator. An obligate irritant agent has not been added because increased epidermal thickness in the case of irritant reactions has already been documented quantitatively by RCM, conventional OCT and high-frequency ultrasound [1, 23, 34] and qualitatively by immunohistochemical findings [22, 23].

### Clinical assessment: (Tables 1, 2)

The allergic patch test reactions were scored by a board-certified specialist of dermatology experienced in reading patch test results, using the visual grading scale recommended by the International Contact Dermatitis Research Group and the North American Contact Dermatitis Group [33]. Irritant reactions were scored according the irritant reaction types proposed by Wahlberg et al. [33].

### Cross-sectional (CS) and en-face (EF) imaging by high-definition optical coherence tomography

High-definition optical coherence tomography (Skintell^®^ Agfa Healthcare Belgium) uses a combination of parallel time-domain interferometry and adaptive optics resulting in a constant homogenous high resolution of 3 µm in all directions and dimensions, allowing visualization of cells in the epidermis, papillary dermis and superficial reticular dermis in their micro-architectural context at up to 570 µm in depth. A detailed description of the HD-OCT has been published [4–7].

Relevant RCM features [3] for diagnosis of ACD and ICD were studied on the EF HD-OCT images (Table 3). This set of morphological features described for RCM is very similar to those described for HD-OCT [7]. In contrast to RCM, the in-buildt HD-OCT slice (CS) mode identifies the exact 3-D position of the selected EF image.

**Table 3:** Relevant RCM features for diagnosis of ACD and ICD respectively

RCM feature	ACD		ICD	
	2d°	4d°	2d°	4d°
Superficial epidermal changes				
Stratum corneum disruption/detached corneocytes	(−/+)	(−/+)	(+++)	(++)
Parakeratosis	(+/−)	(+/−)	(+++)	(++)
Spongiosis^a^ of SG	(++)	(+++)	(++)	(+)
Spongiosis of SS	(++)	(+++)	(++)	(+)
Exocytosis SG	(++)	(+++)	(++)	(+++)
Exocytosis SS	(++)	(+++)	(++)	(+++)
Vesicle formation^b^ SG	(++)	(+++)	(+)	(+)
Vesicle formation SS	(++)	(+++)	(+)	(+)
Necrosis	(+)	(+)	(+++)	(+++)
Increased epidermal thickness	(−/+)	(+)	(++)	(+++)
Basal layer brightness	(−/+)	(−/+)	(++)	(+)
Blood vessel dilatation	(+)	(++)	(+)	(+)
Superficial dermal inflammatory infiltrate^c^	(+)	(++)	(+)	(++)

Parakeratotic changes are seen as highly reflective polygonal cells at the level of SC with a central dark or bright nucleus

Intradermal necrosis seen as circumscribed dark spaces with irregular borders with detached KC

*d*°days after patch test placing

^a^Depending on the severity of the clinical reaction: mild, moderate or marked spongiosis

^b^Vesicle formation may be microfocal, diffuse or widespread

^c^Inflammatory infiltrate either as aggregates or as single cells

High-Definition Optical Coherence Tomography was performed on the patch test, control patch test and unaffected clinically normal looking paralesional skin. The generated images were analyzed with respect to the following criteria: visualization of individual cells in the stratum corneum, stratum granulosum, stratum spinosum and morphology of dermo-epidermal junction, papillary dermis and superficial reticular dermis.

**Fig. 1:**
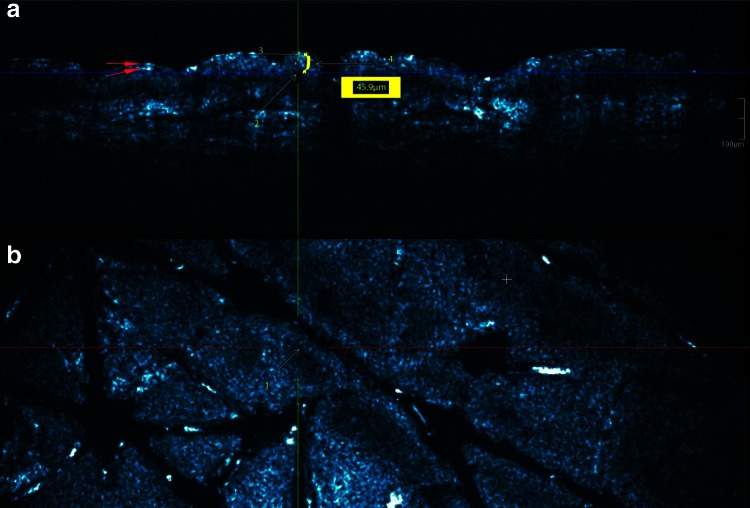
Epidermal thickness (ET) measurement procedure by High-Definition Optical Coherence Tomography 3-D imaging: (**a**) Cross-sectional (CS) mode and (**b**) En-face (EF) mode. Each voxel of the 3-D HD-OCT imaging is defined by a unique set of *x*, *y*, *z* coordinates. (1) First the basal cell layer at the suprapapillary plate will be searched for in the EF mode at the cross of *green* and *red line* (*yellow arrow* “1”). (2) The exact position of the basal cell layer in the CS mode can be determined at the cross of *green* and *blue line* (*yellow arrow* “2”). (3) Stratum corneum is defined as a hyporeflective “band” limited by two *thin hyperreflective lines* (*red arrows*). The *upper* one corresponds with the entrance signal and the *lower* one corresponds with an interference signal due to the difference in refraction indices between stratum corneum and stratum granulosum. If stratum corneum is *thin* both *hyperreflective lines* merge to become one *thicker line* (*yellow arrow* “3”). (4) Epidermal thickness is then measured between this *thicker line* and the *blue line* by using the measurement toolbar of the Skintell(R)program (*yellow arrow* “4” pointing to the *yellow accolade*)

Epidermal thickness (ET) measurements have been performed using the measurement toolbar of the Skintell^®^ program. Each voxel of the 3-Dimensional HD-OCT image is defined by a unique set of *x*, *y*, *z* coordinates. These coordinates permit to measure with great accuracy in vivo and real time the epidermal thickness three-dimensionally and non-invasively. The ET measurement was assessed at the suprapapillary plate in between adnexal epithelium on three different spots on CS image: one with the visual highest ET, one with the visual lowest ET and one spot with intermediate ET. The measurement procedure is demonstrated in Fig. 1. Measurements were carried out in a climate-controlled room, at 24 °C and 40 % relative humidity. On each of the three individual spots ET measurement has been repeated three times. The average of the nine measurements was calculated corresponding to the mean ET. All HD-OCT measurements were performed by the same investigator.

### Statistical analysis

Numerical variables such as epidermal thickness measurements were analyzed using the independent two-sample *t* test or Welch’s *t* test for unequal sample size and unequal variances. The same parametric procedure was used to evaluate the optimization of the visual patch test scoring by HD-OCT. Pearson’s correlation coefficient between clinical scores and selected HD-OCT features was determined.

## Results

### Visual patch test scoring at first and second reading (Table 2)

The overall irritative/allergic reactions ratio was 26/20 at the first visual reading and 28/20 at the second visual reading. The individual irritative/allergic reactions ratio for each transition metal was 10/3(Cr), 10/2(Co), 4/10(Ni) and 2/5(Pd) at first reading and 8/1(Cr), 8/3(Co), 4/12(Ni) and 8/4(Pd) at second reading.

### Normal looking skin assessed by HD-OCT (Fig. 1)

Cross-sectional and EF HD-OCT features of normal skin were similar as those described previously [4]. In the CS mode stratum corneum was defined as a hyporeflective “band” limited by two thin hyperreflective lines. The upper one corresponded with the entrance signal and the lower one corresponded with an interference signal due to the difference in refraction indices between stratum corneum and stratum granulosum. If stratum corneum was very thin both hyperreflective lines merged to become one thicker line. The epidermis was seen as a more reflective homogenous band below the stratum corneum. The sub-basal membrane area appeared as a dark zone in between the basal cell layer and the higher reflective papillary dermis. On EF mode the corneocytes and the grainy cells were identified. The typical honeycomb structure of the stratum spinosum cells could be noticed. The basal cells around the dark dermal papillae could be observed. Adnexal structures and blood vessels were differentiated from other dermal microstructures.

### HD-OCT features of allergic contact dermatitis (Figs. 2, 3, 4, 5)

Cross-sectional and EF HD-OCT features of ACD were similar as those described previously for acute spongiotic dermatitis [7]. Darker areas relative to the surrounding epithelium of the stratum spinosum corresponding to spongiosis were seen on EF HD-OCT images. In these areas larger intercellular spaces between round to polygonal mildly reflective keratinocytes were observed. On CS HD-OCT images an increase in epidermal thickness but decrease in reflectivity could be observed. Smaller but higher reflective cells, single or in small aggregates were observed at the level of the stratum spinosum in both EF and CS modes. Intercellular edema stretching apart keratinocytes sometimes resulted in the formation of intraepidermal vesicles. This vesicle formation secondary to focal areas of spongiosis could be noticed on both EF and CS modes. These vesicles presented as dark round to poly-lobulated areas. In case of severe allergic reactions macro-vesicles were observed. Cells could be observed in- and outsides these cavities.

**Fig. 2:**
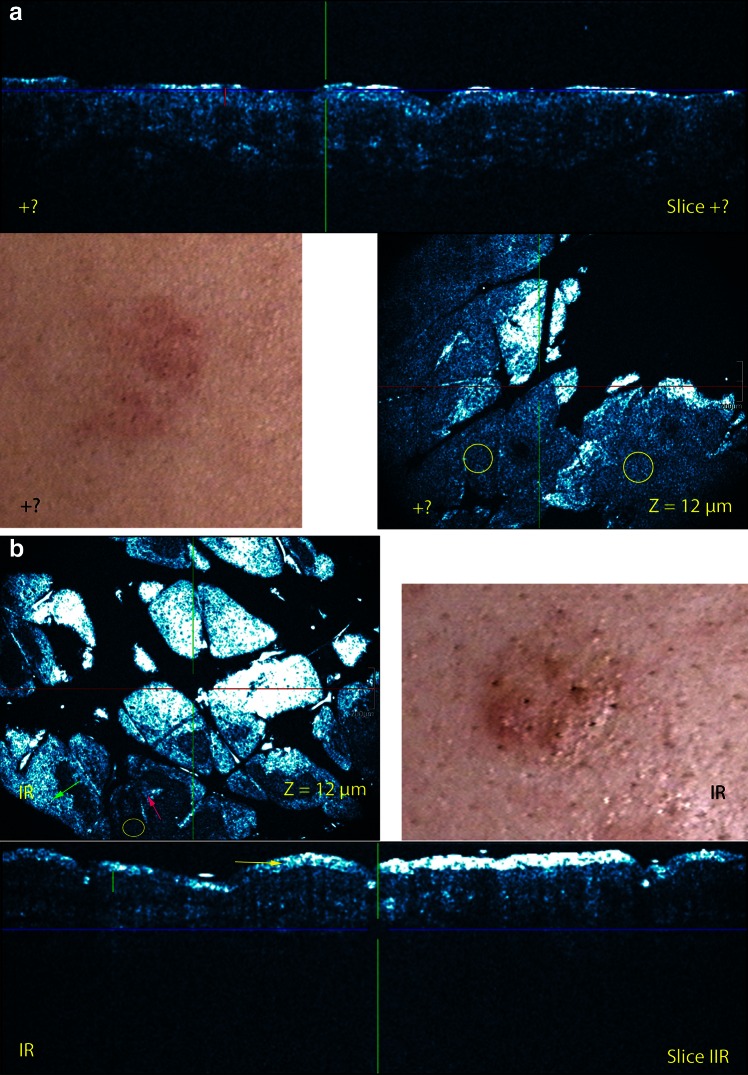
Visual patch test scoring graded and corresponding HD-OCT images 48 h after patch application: (**a**) +? for Ni(II) and (**b**) IR for Cr(VI).On the corresponding CS (slice) HD-OCT imaging a difference in epidermal thickness above the suprapapillary plate is observed between both conditions (*green* versus *red vertical line*). On the IR CS HD-OCT image a disruption of the stratum corneum is noticed (*yellow arrow*). On the IR EF image a parakeratosis (*green arrow*) is noticed as well as several necrotic keratinocyte (*magenta arrow*). On EF images of both conditions spongiosis is present (*yellow circles*). *Z*-values = depth in µm of the en-face image

**Fig. 3:**
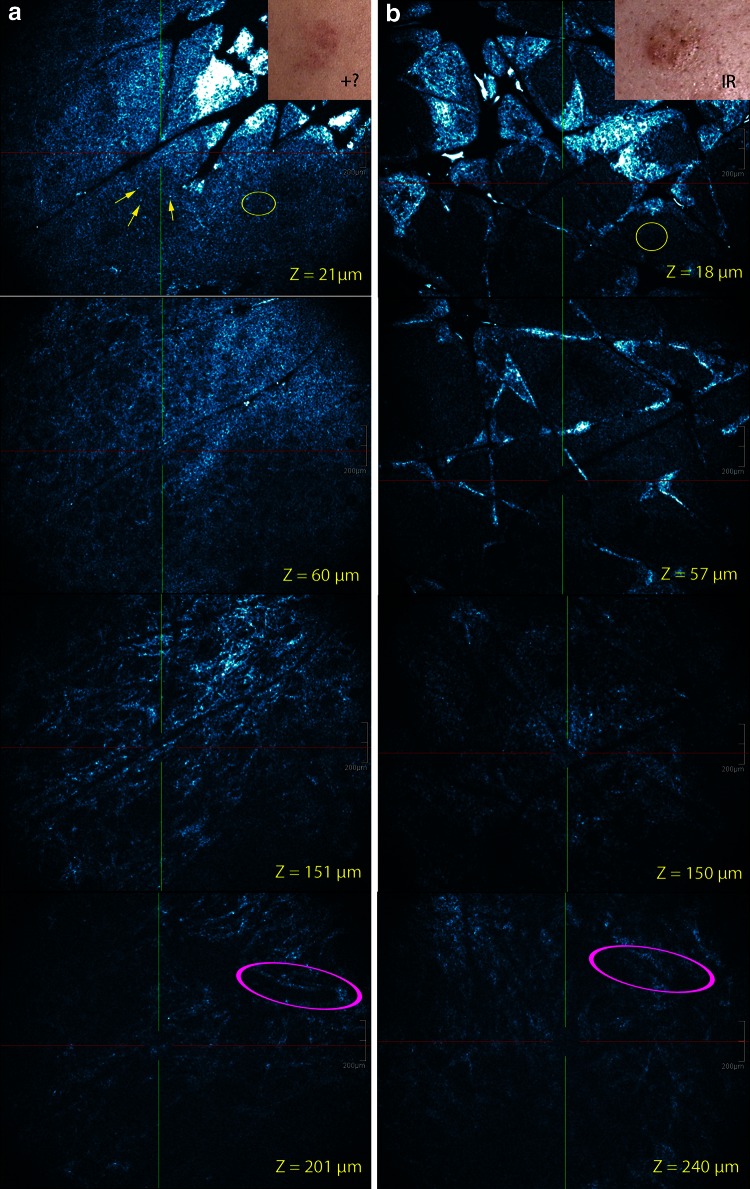
Visual patch test scoring graded and corresponding EF HD-OCT images 48 h after patch application: (**a**) +? for Ni(II) and (**b**) IR for Cr(VI). Pronounced spongiosis of stratum granulosum and stratum spinosum is observed in both reaction types (*yellow circles*). No vesicle formation is noticed. Exocytosis in stratum granulosum and stratum spinosum is more pronounced in the +?-reaction (*yellow arrows*). Blood vessel dilatation and superficial dermal inflammatory infiltrate is similar in both reaction types (*magenta circles*). Dermal edema is almost absent because dermal structures are visible at higher *Z*-values. *Z*-values = depth in µm of the en-face image

**Fig. 4:**
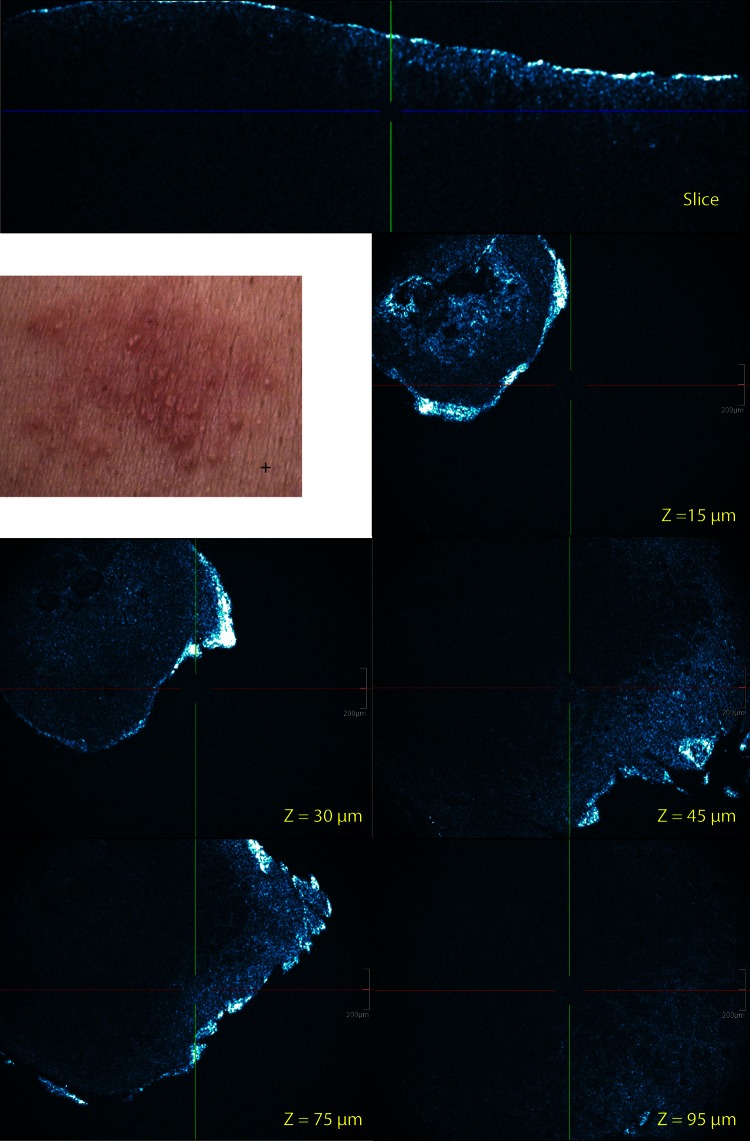
Visual patch test scoring and corresponding cross-sectional and EF (at different *Z*-values) HD-OCT images of Ni (II) allergic reaction graded (+) 48 h after patch application. Spongiosis and dermal edema is more severe compared to +? reactions. Intraepidermal visicle formation is observed. Due to dermal edema the visualization of dilated blood vessels and superficial dermal inflammatory infiltrate is lesser. *Z*-values = depth in µm of the en-face image

**Fig. 5:**
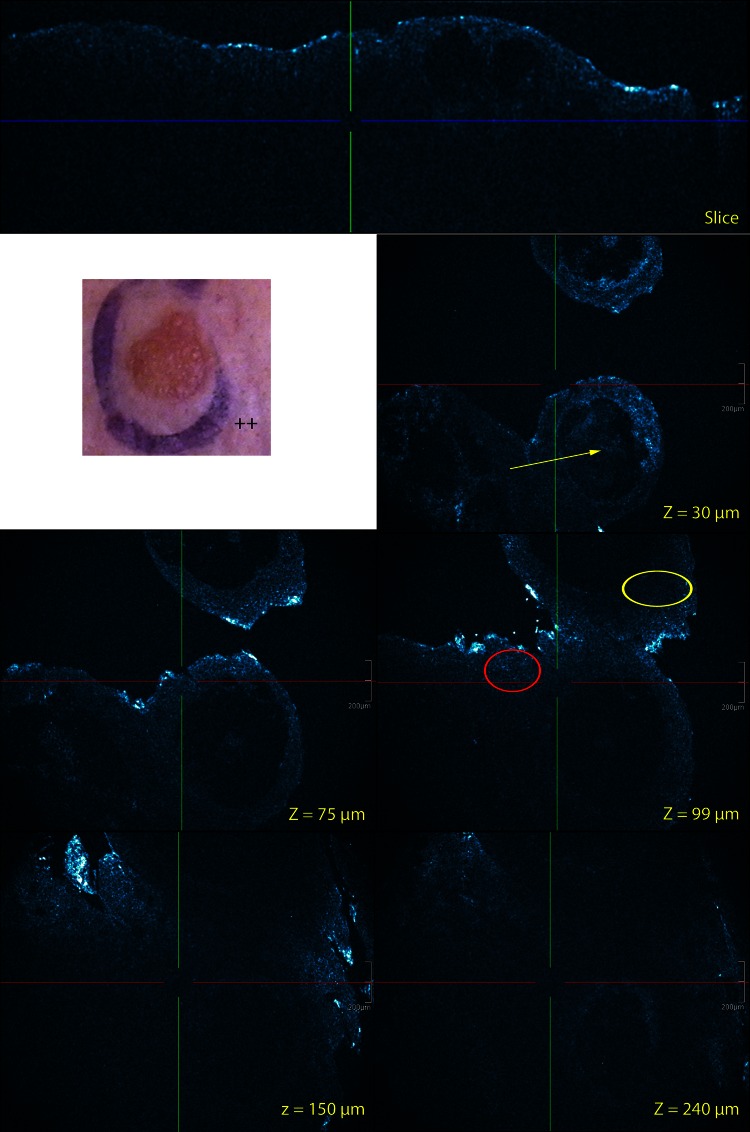
Visual patch test scoring and corresponding CS and EF (at different *Z*-values) HD-OCT images of Cr(VI) allergic reaction graded (++) 48 h after patch application. The more severe the reaction the more intense the spongiosis (*yellow circle*), vesicle formation (*yellow arrow*) and exocytosis (*red circle*). The more severe the dermal edema the lesser the blood vessel dilatation and superficial dermal inflammatory infiltrate could be perceived. *Z*-values = depth in µm of the en-face image

Edema in the dermis appeared to lower dermal reflectivity. In severe forms no dermal reflectivity remained. In moderate forms, dilated signal free cavities in the dermis corresponding to blood vessels were noticed. In these forms the dermal perivascular inflammation was marked with small highly reflective cells.

### HD-OCT features of irritant contact dermatitis (Figs. 2, 3)

A significant increase in epidermal thickness (ET) was found in ICD by CS HD-OCT measuring the thickness of the suprapapillary ET.

This increase was significant compared to doubtful positive (+?) ACD and normal skin at the first reading (*p* < 0.001) and second reading (*p* < 0.001) (Welch’s *t* test, unequal sample sizes and unequal variances). The ET of normal skin of the back was 47.38 µm [+/−1.07 µm; 95 % confidence interval (CI)]. In ICD at first reading, the ET increased with 12.57 µm (+/−2.48 µm; 95 % CI) compared to 2,51 µm increase (+/−3.71 µm; 95 % CI) in doubtful positive (+?) ACD. At second reading, the ET increases in ICD measured 9.87 µm (+/− 2.33 µm; 95 % CI) and in doubtful positive (+?) ACD 2.89 µm (+/− 2.56 µm: 95 % CI).

Superficial epidermal morphological and cellular changes were also found in ICD compared to ACD. These changes could be observed in combined (3-D) CS and EF HD-OCT mode. The changes consisted of (1) significant parakeratosis whereby parakeratotic cells appeared as bright highly reflective round to oval nucleated structures centrally located within stratum corneum, (2) stratum corneum disruption with disrupted corneocytes presented as single or sheets of multiple discrete mildly bright cellular structures larger than normal keratinocytes without identifiable nucleus, (3) necrotic keratinocytes presented on HD-OCT as polygonal structures brighter and larger than surrounding keratinocytes.

### HD-OCT features: correlation with clinical/visual severity. Grading of inflammation by HD-OCT

Two groups of features describing inflammation could be determined. The first group consisted of features linked to water accumulation: spongiosis, intraepidermal vesicle formation and dermal edema. The second group consisted of features related to cellular infiltrate: exocytosis and superficial dermal inflammatory infiltrate associated with blood vessel dilatation. The degree of epidermal changes related to water accumulation correlated well with clinical severity scores. The degree of dermal edema also correlated with clinical severity scores. The more severe the dermal edema, the less the blood vessel dilatation and superficial dermal inflammatory infiltrate could be perceived Figs. 4, 5.

### Optimization of the visual patch test scoring by HD-OCT

Table 2 displays the HD-OCT assisted patch test scoring at the first and second reading. The difference was not significant between visual scoring and HD-OCT evaluation at the first reading. HD-OCT assisted scoring at the second reading, however suggested that five irritant reactions could be re-classified as allergic reactions and two doubtful reactions as irritant reactions. At second reading, reactions to Pd were clinically most often misclassified as IR instead of doubtful (+?) reactions. Although HD-OCT improved patch test scoring, the difference between visual scoring and HD-OCT assisted scoring was not significant.

## Discussion

Differentiating ACD and ICD remains a challenge for clinicians and researchers [26]. A perfect golden standard permitting differentiation between ACD and ICD does not exist.

Both conditions can be clinically and histologically indistinguishable despite their different pathogenesis [17, 28, 35]. However, two hallmarks of ICD are perturbation of the skin barrier and the epidermal regenerative hyperproliferation [9, 25]. The predominant ultrastructural change in the epidermis of acute ACD is spongiosis [20].

This paper suggests that the increased epidermal thickness (ET) observed in ICD at first reading is a significant finding useful in differentiating doubtful (+?) ACD from (IR) ICD reactions, following exposure to transition metals. The increased ET is probably the result of acute regenerative hyperproliferation and disturbed differentiation expressed by parakeratosis. [9, 28] Normal values however, have to be established for reference when direct comparisons are not made in an experimental set-up. The increased ET in the case of irritant reactions has already been documented quantitatively by RCM, conventional OCT and high-frequency ultrasound [1, 23, 34] and qualitatively by immunohistochemical findings [22, 23]. Conventional OCT has been used for the quantification and monitoring of the epidermal and dermal changes in irritant contact dermatitis. Repeated measurements were performed in healthy volunteers after experimental induction of irritant contact dermatitis. A thickening of the epidermis was a constant finding and could be monitored over time [34].

Furthermore, this paper suggests that HD-OCT might be an useful and unique tool for non-invasive in vivo real-time three-dimensional ET measurements. In vivo measurements of ET have been systematically studied in healthy skin using RCM [27] and conventional OCT [12, 13, 18, 32]. Epidermal thickness measurement by RCM is based on at least one vertical (*z*-axis) stack of 21 EF images collected at 5 µm intervals form the skin surface to a depth of at least 100 µm. The ET corresponds to the distance from the first living cells of the granular layer to the basal cells lying on the top of the dermal papillae. Although some epidermal properties vary by site, the most important limitation is the degree of within-site variation accounting for between 50 and 74 % of the total variation in ET observed. This variation was not due to measurement errors but corresponds to topographical irregularity. This fine-scale variation limits the use of RCM for quantitative studies of epidermis [27].

Conventional optical coherence tomography is an interesting tool for ET measurements. Epidermal thickness is determined in the OCT image (B-scan) on the computer screen using the integrated measure tool (ruler). ET is manually measured in the OCT image from the skin surface reflection (entrance signal) to the first well-demarcated change of reflectance intensity as expressed in a more signal-poor zone [13]. Local positioning of the dermo-epidermal junction between observers was often different. Therefore, a tendency to overestimate the thickness of the epidermis was noticed [12].

High-definition optical coherence tomography offers some practical advantages in ET measurement by providing three micrometer lateral and axial resolution data in the 3D micro-anatomical context [4–7]. EF HD-OCT imaging permits to define with precision both the position of the junction between stratum corneum and granular layer and the position of the basal cell layer in contact with the dermo-epidermal junction. Once both positions have been determined ET measurement can be performed on CS HD-OCT imaging with higher precision than conventional OCT because of the higher axial resolution of the former (3 µm compared to the 7.5 µm axial resolution of OCT). In a recent paper, HD-OCT measurement of ET in relatively thin lesions appears to create valid results compared with routine histopathology indicating that this method could be well suited to skin monitoring purposes and therapy outcome assessments [14].

A previous study suggested that HD-OCT features of inflammatory skin conditions with epidermal alteration correlate well with dermatopathologic descriptors as defined in RCM [7]. The morphological CS and EF features of acute spongiotic skin conditions imaged by HD-OCT in this study were very similar to those demonstrated for conventional OCT and RCM, respectively [11, 16].

Reflectance confocal microscopy has been used in non-invasive in vivo studies to demonstrate intraepidermal and superficial dermal cellular changes and micro-architectural alterations in ACD and ICD. These features were later confirmed by correlating them with histopathology [16]. Furthermore, the kinetics of ACD and ICD over time, as well as the individual ethnic susceptibility to ICD have also been examined by using RCM [1, 2]. The sensitivity and specificity of a series of 12 RCM criteria to differentiate ACD and ICD in reference to patch testing have been determined [3].

This study demonstrates that the degree of changes in some epidermal HD-OCT features of acute spongiotic skin conditions, such as spongiosis, vesicle formation and exocytosis, correlates well with clinical severity scores confirming the findings of previous histopathological studies and RCM studies [1, 3, 36]. Furthermore, the degree of changes in dermal HD-OCT features correlates also with clinical severity scores. Dermal edema correlates with clinical severity scores which was clearly observed in the CS HD-OCT images. The more severe the dermal edema, the less the blood vessel dilatation and superficial dermal inflammatory infiltrate could be perceived. This inflammatory infiltrate in HD-OCT is very similar in ICD and (+?) or (+) ACD reactions which is in agreement with the literature [21].

The overall irritative/allergic reactions ratio was not significantly different between visual scoring and HD-OCT evaluation. At second reading, reactions to Pd were most often misclassified as IR instead of doubtful (+?) reactions. The morphology of patch test reactions can vary with different metals. Some allergens such as Pd report to show a delay in the development of positive tests [15]. This could probably explain why Pd “behaves” as Cr/Co at first reading with high irritative/allergic reactions ratio and as Ni at second reading with low irritative/allergic reactions ratio. The allergic reactions to Pd are significantly more present at second reading in this study. At first reading we probably observe indirectly the stimulation of innate immunity by Pd [24]. As the strength of the innate inflammatory response is much higher for Ni than for Pd, the threshold for activation of the adaptive immune system is eventually much higher for Pd [15]. As a result, it takes more time before an allergic reaction to Pd develops.

We found an optimization of the visual patch test scoring by HD-OCT assessment although not significant. A possible explanation could be the experience in visual patch test scoring. Dermatologists not so familiar with patch test reading could be assisted by HD-OCT to increase their diagnostic accuracy. The qualitative and quantitative differences found between the reactions may imply that earlier readings of patch tests may be possible if supplemented by HD-OCT. HD-OCT assisted patch test scoring could be very useful when it is difficult to assess erythema; e.g. when dealing with patients with Fitzpatrick phototype IV or higher [2]. Additional studies are essential to validate these suggestions.

Some important drawbacks need to be mentioned. Firstly an obligate irritant agent has not been included into the test substances because the increased ET in the case of irritant reactions has already been documented previously by other authors [1, 22, 23, 34]. In addition, the use of transition metals was thought to reflect a real-world situation better. Secondly the visual patch test scoring as well as the HD-OCT assisted patch test scoring was performed only by one observer. However, as mentioned in the method section on three different spots in between adnexal epithelium and at the suprapapillary plate, ET measurement has been repeated three times The average of the nine measurements was calculated corresponding to the mean ET.

In conclusion, HD-OCT may be considered as a unique tool for in vivo, non-invasive and real time three-dimensional epidermal thickness measurements. Epidermal thickness measurements by HD-OCT help to differentiate IR from doubtful (+?) reactions in patch testing and may be performed repeatably in real time. Selected HD-OCT features corresponded well with severity of visual scoring. These features might help to quantify the degree of inflammation in inflammatory skin conditions. HD-OCT might help in optimizing visual patch test scoring in some situations.
